# Recurrent Colorectal Liver Metastases in the Liver Remnant After Major Liver Surgery—IRE as a Salvage Local Treatment When Resection and Thermal Ablation are Unsuitable

**DOI:** 10.1007/s00270-021-02981-4

**Published:** 2021-11-10

**Authors:** Lea Hitpass, Martina Distelmaier, Ulf P. Neumann, Wenzel Schöning, Peter Isfort, Sebastian Keil, Christiane K. Kuhl, Philipp Bruners, Alexandra Barabasch

**Affiliations:** 1 Department of Diagnostic and Interventional Radiology, University Hospital RWTH Aachen, Pauwelsstraße 30, 52074 Aachen, Germany; 2 Department of Surgery and Transplantation, University Hospital RWTH Aachen, Aachen, Germany; 3 Department of General, Visceral and Transplant Surgery, Charité, Berlin, Germany

**Keywords:** Irreversible electroporation (IRE), Colorectal liver metastases (CRLM), Recurrence, Safety, Efficacy

## Abstract

**Purpose:**

To examine the safety and short-term oncologic outcomes of computer-tomography-guided (CT-guided) irreversible electroporation (IRE) of recurrent, irresectable colorectal liver metastases (CRLM) after major hepatectomy deemed unsuitable for thermal ablation.

**Patients and Methods:**

Twenty-three patients undergoing CT-guided IRE of recurrent CRLM after major hepatectomy were included in this study. All tumors were located adjacent to sole remaining intrahepatic blood vessels and bile ducts, precluding thermal ablation. Patients underwent systematic clinical and imaging follow-up, including magnetic resonance imaging of the liver at 1-month and 3-month intervals thereafter. Time to local and intrahepatic tumor progression within 12 and 36 months and associated risk factors were assessed using Kaplan Meier and Cox regression analysis, respectively.

**Results:**

Complete ablation with a safety margin of at least 0.5 cm was achieved in 22/23 (95.6%) patients. No vessel injury or thrombosis occurred. Five patients developed moderate biliary stenosis after a median of 4 weeks, without requiring treatment. Local tumor-progression-free rates within 12/36 months were 64%/57.4%, respectively. Intrahepatic-progression-free rate within 12/36 months was 36.4%/19.5%, respectively. Five (23%) patients were tumor-free at the end of follow-up. Multivariate Cox regression analysis did not show any association between local tumor-progression-free rates and patient age, target tumor size, primary tumor side or synchronicity of liver metastases.

**Conclusion:**

In this highly selected patient population with local recurrences of CRLM after major surgery, IRE was shown to be a safe salvage treatment option when thermal ablation is unsuitable.

## Introduction

The comparison of surgical resection vs. image-guided local ablative therapies for the treatment of colorectal cancer liver metastases (CRLM) is a current subject of research [1, 2]. In this regard, local ablative treatment has been associated with good local tumor control [3, 4] and promising overall survival rates [5, 6].

Although the treatment of colorectal liver metastases (CRLM) is a multidisciplinary challenge, surgery is the standard treatment approach for patients with resectable oligometastastic colorectal cancer, according to ESMO and NCCN guidelines [7, 8]. However, these guidelines also acknowledge the emerging evidence supporting the application of local ablative therapies and recommend these as an alternative option, either alone or in combination with surgery [7, 8]. Prerequisites for these recommendations vary between these guidelines: For NCNN, all sites of metastatic spread must be amenable by these methods [8], whereas for ESMO metastases must be in unfavorable locations or considered oligometastatic disease [7].

Furthermore, local tumor recurrence after extended liver surgery is often not amenable to repeated resection. Generally, this is due to either insufficient volume of the future liver remnant (FLR) with associated risk of posthepatectomy liver failure (PHLF) or “small-for-size” syndrome (SFSS) (a complex of symptoms often caused by postsurgical portal hypertension [9–11]) or because of the tumor recurrence’s vicinity to the—often singular—remaining blood vessels and/or bile ducts. In the latter, thermal local image-guided ablation is not a viable option, as thermal injury or thrombosis of the remaining vessels and bile ducts represents a relevant risk and proximity to veins and/or bile ducts has been associated with suboptimal ablation [12]. Therefore, a non-thermal ablation technique like irreversible electroporation (IRE) may be a safe and viable treatment option for centrally located liver tumors with margins adjacent to major bile ducts [13–16], which still ensures complete ablation with a safety margin, a pre-requisite for long-term tumor control [17–20].

We report on our experience regarding the efficacy and safety of computed tomography (CT)-guided IRE in patients with recurrent CRLM after extended liver resection, not amenable to surgical excision or thermal ablation.

## Material and Methods

An IRB-approved prospective longitudinal observational study was conducted, at an academic hepatobiliary cancer center (internal reference number EK 071-21).

### Patient Cohort

From June 2012 to August 2019, all consecutive patients undergoing percutaneous CT-guided IRE for recurrent CRLM after extended surgical resection, who were unsuitable for thermal ablation, were included in this study.

Operations included major liver resections (such as hepatectomies, trisectionectomies or extensive non-anatomical resections), carried out in curative intent.

### Treatment Decision

The decision for IRE was made by a multidisciplinary tumor board consisting of hepatobiliary surgeons, gastroenterologists, oncologists, radiation therapists, nuclear medicine specialists, and interventional radiologists, based on the following criteria:

- Irresectable CRLM: All patients underwent surgery to a maximum extent and were regarded as unresectable following the recommendation of Clavien et al. [21]
- Up to three liver metastases, each less than 3 cm in diameter
- Absence of prognostically relevant extrahepatic tumor burden
- Preserved liver function reflected by normal levels of:
- Bilirubin (< 2.0 mg/dL)
- Albumin (> 3.0 mg/dL)
- Blood coagulation status along CIRSE (cardiovascular and interventional radiological society of Europe) guidelines for interventional procedures (Quick > 50%, prothrombin time (PTT) < 50 s., thrombocytes > 50,000)
- Eastern Cooperative Oncology Group (ECOG) performance status < 2

Following the consensus guideline by Ruarus et al. [14], IRE was preferred over thermal ablation if liver metastases were < 10 mm from the postoperatively remaining segmental or lobar portal vein branches, the corresponding bile ducts and/or main (left, middle, right) hepatic veins (see Table 1).

**Table 1:** Target tumor localization

Patient demographics of all 23 patients	
Age, y (mean, SD)	60 ± 11
Gender (M, F)	15: 8
Mean tumor size (mm, range)	15 (4–39)
*Target tumor localization*	*n* = 32
Left hepatic portal vein	13
Right hepatic portal vein	2
Anteromedial portal vein	3
Middle hepatic vein	10
Right hepatic vein	2
Vena cava inferior	2
*Pre-interventional surgery*	*n* = 23
Right trisectionectomy	3
Left trisectionectomy	2
Right hemihepatectomy	1
Bisectionectomy + min. 3 non-anatomical resections	8
Trisectionectomy + non-anatomical resection	2
Right hemihepatectomy + bisectionectomy (S II/III)	7

### IRE Procedure

IRE procedures in our institution from 2021 onwards have been performed by two interventional radiologists with at least 7 years of experience in CT-guided thermal ablations. Interventions were performed under general anesthesia, according to manufacturer recommendations, with patients in supine or left lateral position. Unipolar, 19 Gauge IRE probes (NanoKnife, AngioDynamics, Amsterdam, The Netherlands) with an active tip length of 15–25 mm were inserted in parallel, under CT-guidance. The number of probes used depended on tumor size, shape, and planned margin size. The puncture pathway for needle placement was optimized for preservation of blood vessels and bile ducts, as well as minimal damage to surrounding parenchyma. Correct probe positioning was confirmed by a contrast-enhanced CT scan (Somatom Force, Siemens, Erlangen, Germany) in arterial and venous phases, using a bodyweight-adapted amount of a non-ionic contrast agent (1 mL/kg bodyweight; Ultravist 370, Bayer Schering Pharma AG, Berlin, Germany). Pulse application was then performed, ensuring electrocardiographic gating, with the following parameters: 70 pulses pro probe pair, 90 µs pulse length, 3000 V maximum voltage. Another contrast-enhanced liver CT-scan was acquired on completion, to confirm complete tumor ablation and rule out major procedure-related complications. As reported in previous studies, a sufficient ablation was achieved when the entire target tumor was included and a safety margin of at least 0.5 cm was delimitable [22]. The pre- and postablational portal venous CT images were compared considering anatomic landmarks as recommended in the study by Wang et al. [18] to confirm technical success.

The interventions were performed as inpatient procedures, with patients being kept for at least 24 h on a standard care unit for postinterventional observation. The day after the procedure, a multiphase CT examination was performed to exclude postinterventional complications.

### Pre-Interventional and Follow-up Imaging Protocol

Pre-interventional imaging consisted of gadobutrol-enhanced liver MRI (Gadovist, Bayer Schering Pharma AG, Berlin, Germany) within 2 weeks and contrast-enhanced CT of the thorax and abdomen within 4 weeks pre-IRE.

According to CIRSE guidelines [23] and Ahmed et al. [24], a contrast-enhanced CT of the liver was performed immediately after the procedure to analyze technical success and rule out postinterventional complications; follow-up imaging consisted of liver MRI 4- and 12-weeks post-IRE as well as contrast-enhanced CT of thorax and abdomen 4 weeks post-IRE and 6-monthly thereafter.

Two body radiologists with 14 and 9 years of experience systemically analyzed all images in consensus, according to CIRSE guidelines and quality improvement guidelines by Ahmed et al. [7, 23, 24].

Assessed Parameters were:

- Incomplete ablation, defined as residual scattered, nodular or eccentric periablational enhancement
- Local tumor progression (LTP), defined as appearance of tumor foci at the edge of ablation zone after at least one confirmed complete ablation
- Locoregional tumor recurrence, defined as tract seeding
- New distant intrahepatic tumor growth
- New extrahepatic tumor growth

### Assessment of Complications

Complications were categorized according to Ahmed et al. [24] as major complications that lead to substantial morbidity and disability that increased level of care, or resulted in hospital admission, or substantially lengthened hospital stay. All other complications were considered as minor.

Moreover, complications were subdivided into:

- Immediate (during or up to 24 h after the procedure): e.g. bleeding, pneumothorax, vessel thrombosis, portal vein occlusion, infarction, biloma
- Periprocedural (within 30 days after the procedure): e.g. vessel stricture, thrombosis, infarction, biliary injury or biloma
- Delayed (> 30 days after procedure): e.g. vessel stricture, thrombosis, infarction, bile leak or stricture, biloma.

### Statistical Analysis

Statistical analyses were performed using IBM SPSS Statistics for Windows, version 25.0.0.0 (IBM Corp., Armonk, N.Y., USA), and *p*-values < 0.05 were regarded as statistically significant.

Local progression-free rates and intrahepatic progression-free rates, as well as time to progression, were analyzed using Kaplan–Meier analyses. The association between time to progression and patient- or target-tumor-related variables was analyzed using Cox regression analyses.

## Results

### Patient and Target Lesion Characteristics

Overall, 23 patients (15 male; 60 ± 11 years) with 32 CRLM were included. Eighteen patients had left-sided colon cancers, and 83% of all patients (19/23) suffered from synchronous metastatic disease. Median follow-up time was 25 months (range 6–93 months).

Details on pre-interventional surgery and target localization are shown in Table 1.

### Procedure-Related Complications

**Fig. 1:**
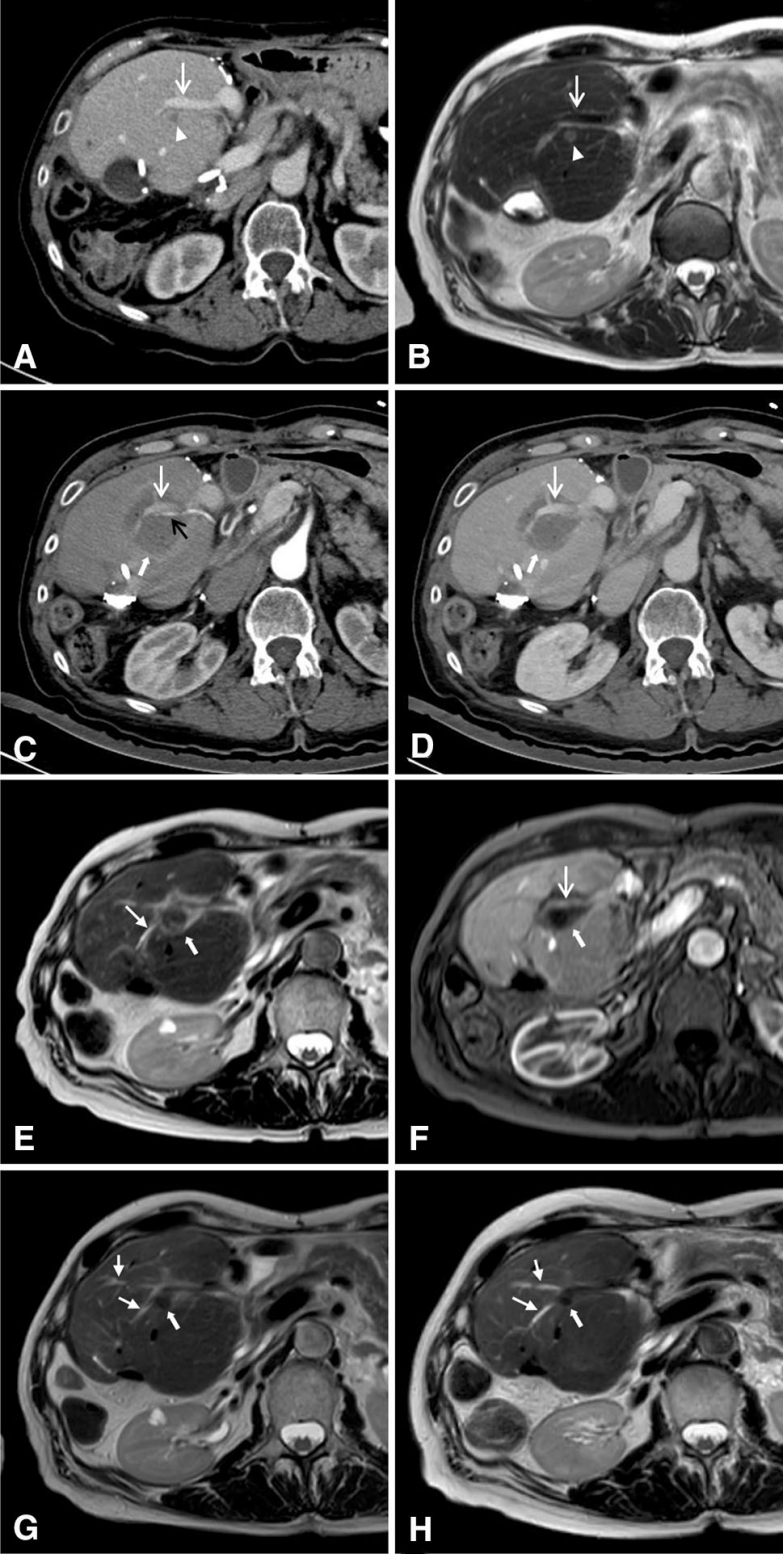
Sample case. 71-year-old female patient with synchronous metastatic colorectal cancer to the liver who priory underwent right hepatectomy and bisegmentectomy of segments II and III. 5 months after surgery the patient was diagnosed with a new metastasis in the liver remnant, which, accordingly, consisted of segments I and IV, only. A multidisciplinary tumor board made the decision to perform IRE. **A** (CT in venous phase) and **B** (MRI: T2w TSE) show the metastases (arrowhead) located immediately adjacent to the sole remaining portal vein branch of segment IV (white open arrow). **C** (CT in arterial phase) and **D** (CT in portal venous phase) show the ablation area immediately after the IRE procedure. The ablation zone (thick arrow) extends beyond the portal vein branch (white open arrow). The adjacent vessels, portal vein branch (white open arrow) and segment-IV-artery (black open arrow) remained perfused. **E** (MRI: T2w TSE) and **F** (MRI: T1w GRE in portal venous phase) show the ablation area 4 weeks after IRE. The ablation zone (thick arrow) has decreased in size. However, the patient now shows evidence of a subsegmental cholestasis (E, white closed arrow) due to an IRE-induced bile duct stricture. Plasma bilirubin remained within normal limits (0.6 mg/dl). The adjacent portal vein branch (white open arrow) is still perfused without evidence of vessel thrombosis. **G** and **H** (MRI: T2w TSE) show the ablation area 3 (**G**) and 6 (**H**) months after IRE. The subsegmental cholestasis (closed arrow) remained stable and did not require treatment (plasma bilirubin 6 months after IRE was 0.42 mg/dl). The ablation zone (thick arrow) further decreased in size. Follow-up imaging did not show any evidence of distant tumor recurrence in and outside the liver. IRE = Irreversible electroporation; CT = computed tomography; MRI = magnetic resonance imaging; T2w = T2-weighted; TSE = turbo spin echo; T1w = T1-weighted; GRE = gradient echo

There were no major complications according to CTCAE and Ahmed et al. [24]. As periprocedural complications, segmental cholestasis due to IRE-related bile duct injury was seen in five (22%) patients on MRI imaging (Fig. 1), during a median follow-up period of 4 weeks (range 0, 14–5 weeks).

### Outcome

**Fig. 2:**
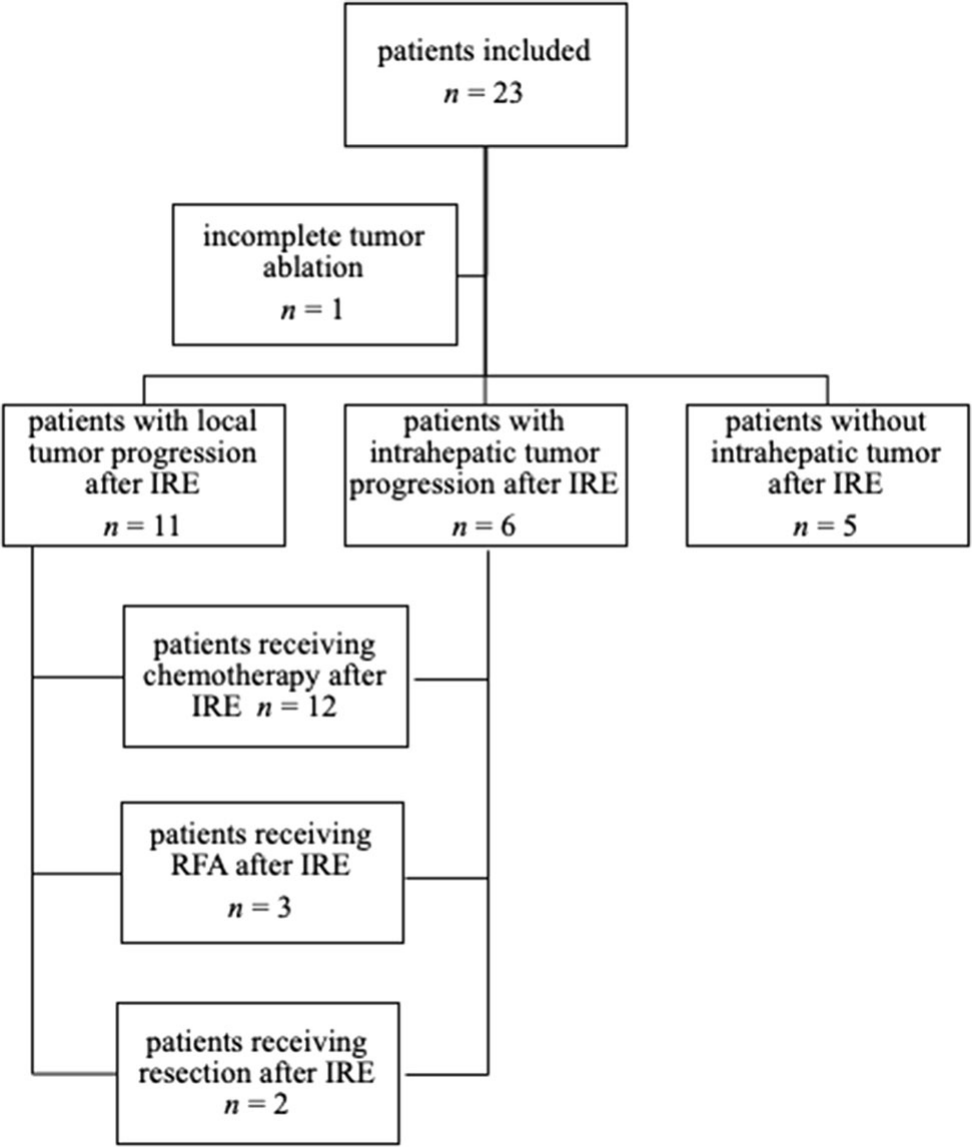
Flowchart–patients follow-up. Overview on patients oncological follow-up, IRE = irreversible electroporation

Post-IRE imaging revealed incomplete target ablation in 1 (4%) patient. Due to extrahepatic tumor progression, no reintervention followed (Fig. 2).

**Fig. 3:**
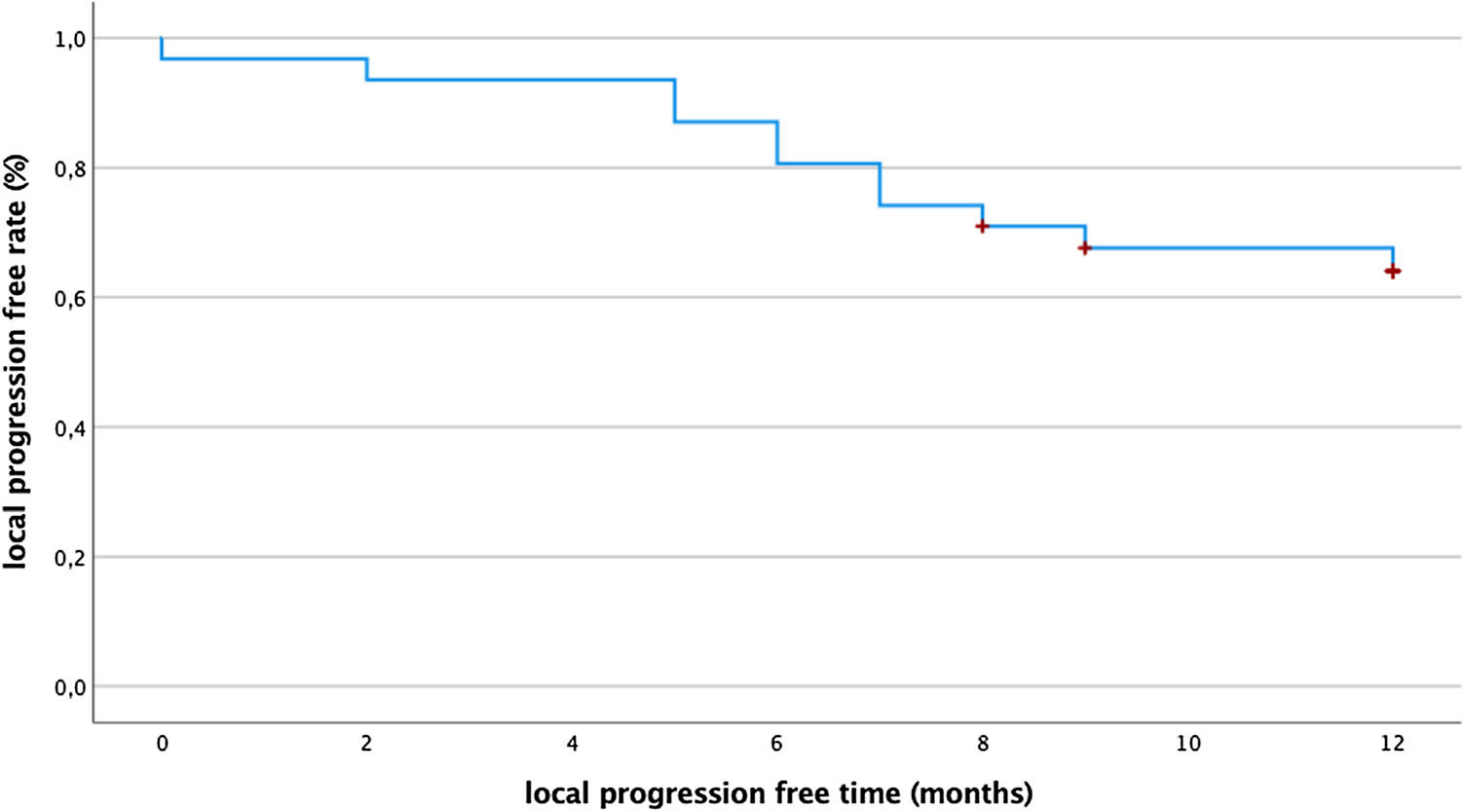
Twelve-month follow-up of local tumor progression

The remaining 22 patients presented 31 lesions directly adjacent to major vessels or bile ducts with a mean diameter of 15.2 mm (range 4–39 mm). Of these, 12/31 (38.7%) lesions presented LTP during follow-up, leading to a 1-year LTP-free rate of 64% and a mean time-to-LTP of 10 months (95% CI 8.6–11.2; see Fig. 3). The 3-year LTP-free rate was 57.4%, resulting in a mean time-to-LTP of 25 months (95% CI 19.6–29.7). All in all, 4/22 (18%) patients suffered from tract seeding.

**Fig. 4:**
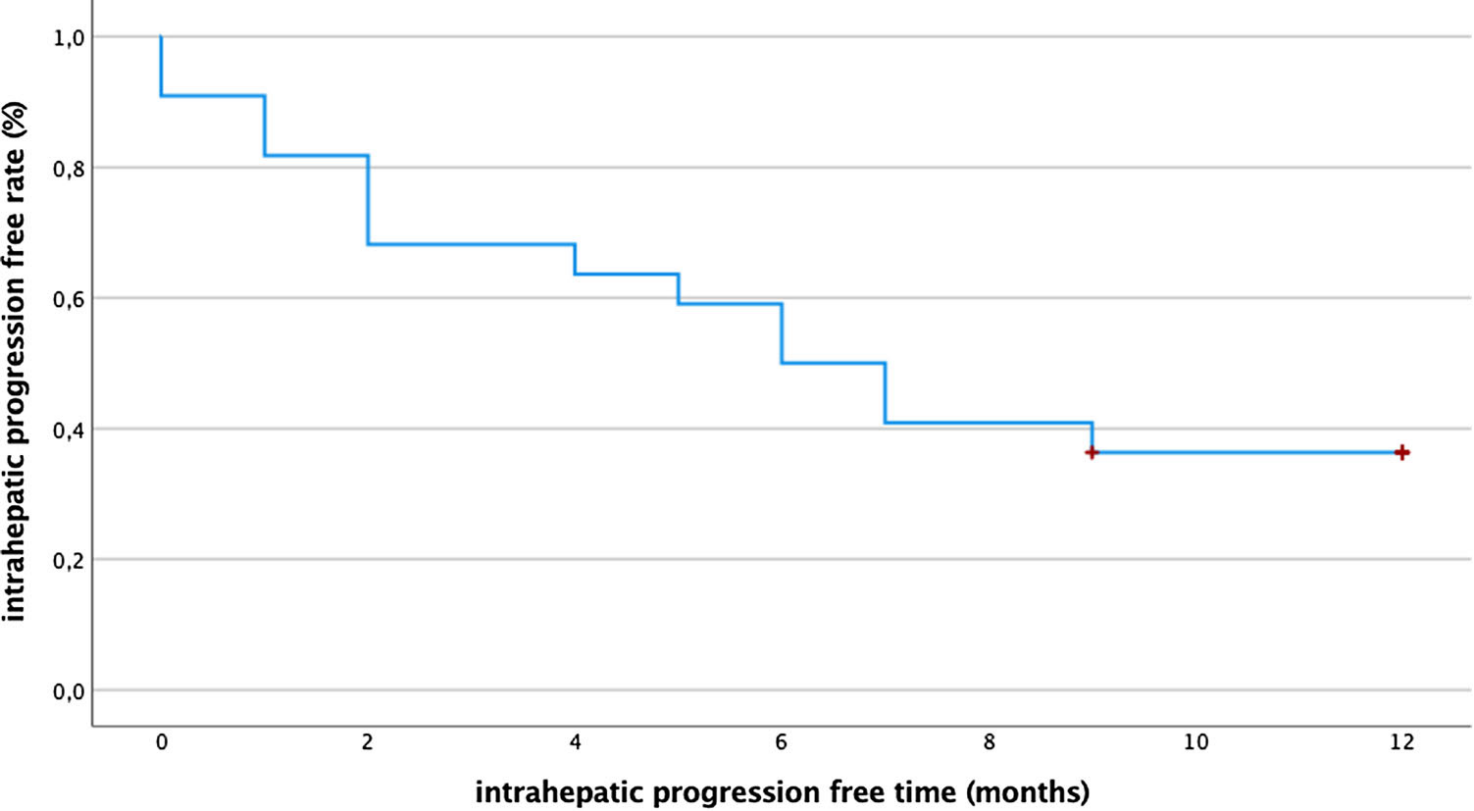
Twelve-month follow-up of intrahepatic tumor progression

Out of 22 patients, 17 (77%) presented local or intrahepatic tumor progression during follow-up and 5/22 (23%) were intrahepatic tumor-free. Intrahepatic tumor control rate was 36.4% within 12 months (intrahepatic progression-free time 7 months (95% CI 5–9); see Fig. 4) and 19.5% within 36 months (intrahepatic progression-free time 13 months (95% CI 8–19)).

Out of 17 patients with intrahepatic tumor progression, 12 (70%) received chemotherapy in palliative intent, three (18%) patients were eligible for radiofrequency ablation, and two (12%) patients underwent resection.

At the end of follow-up, three out of six patients without hepatic metastasis developed pulmonary metastatic disease and three patients remained without any metastasis until end of follow-up (see Fig. 2).

Multivariate cox regression analysis did not show any association between LTP-free rates and patient age, target tumor size, primary tumor side or synchronicity of liver metastases.

## Discussion

Promising overall survival rates of patients with CRLM treated with thermal ablation techniques [4–6] led to an ongoing debate about the comparison of surgical with non-surgical disease management [2, 7, 25]. Still, reaching complete thermal ablation remains a challenge in patients with tumors in the vicinity of large blood vessels, because of the “heat-sink effect” [6, 23, 26]. In these cases, non-thermal ablation techniques like IRE might be a safe treatment option [13–15]. Patients after major hepatectomy usually present with a single portal vein branch and main bile duct, as well as only one (or at most two) major liver veins. Damage to these structures, namely thrombosis or stricture, which has been reported after thermal ablation, bears the risk of acute liver failure (and, as an extension, death) and should therefore be avoided. Accordingly, (a) to reach complete ablation and (b) minimize patient risk, non-thermal ablation techniques such as IRE seem to be the ideal treatment option for these patients.

In this study, CT-guided IRE of recurrent liver metastases after major hepatic resections was performed as a local salvage treatment. A multidisciplinary tumor board deemed all tumors irresectable due to direct proximity to sole remaining blood vessels and/or bile ducts.

No major complications were observed in the study cohort. However, 22% of patients (5/23) developed moderate segmental cholestasis after the procedure. This is lower, but still comparable with previous studies, reporting cholestasis rates of 29–55% after IRE [13, 27]. Since segmental cholestasis was observed after a median time of 4 weeks (range 0–5 weeks) direct injury due to probe placement and tissue-destructive effects of IRE procedure are equally likely. However, no patients required further treatment.

Moreover, although it is known that the application of IRE is capable of inducing considerable heating in the liver and pancreas that is sufficient to cause thermal tissue damage [28], and there are studies that report even higher complications compared with thermal ablations [29], no blood vessel damage, narrowing or thrombosis was observed in our cohort.

In the present study, overall LTP occurred in 38% of patients during a median follow-up period of 25 months. The vast majority of these (92%; 11/12) were noted within the first year after IRE procedure leading to a LTP rate of 36% within 12 months. Although literature suggests that IRE is not as effective as thermal ablation [26], our results are quite comparable with 1-year LTP rates (33–65%) reported in previous studies on RFA [3, 30, 31].

However, it has been shown that the creation of a uniform 5 mm margin around the tumor in every direction is essential when applying RFA, to achieve effective local tumor control. This can decrease the LTP rate to as low as 15% within the first year after ablation [3, 18]. Still, the same study also identified prior hepatectomy as a risk factor for LTP, limiting comparability with our patient cohort, consisting of patients after major hepatectomy only. Nonetheless, further research to evaluate the impact of margin size in preventing LTP after IRE is needed.

Up to now, there is only limited evidence on LTP rates after IRE ranging from as low as 6% after a short-term follow-up of 6 months to 21% after 1 year in the COLDFIRE-2 trial [17, 32]. Notably, these are also lower than most LTP rates reported after RFA and stand in contrast to the assumption that IRE is less effective compared to thermal ablation. Further investigation regarding the efficacy of IRE compared to RFA is needed. Possible explanations for the comparably high LTP rate in our study might be that both previous studies included both patients with and without prior liver surgery and the study that reported a LTP rate of 6% included mainly patients with very small tumors (60% < 1 cm) of different entities, whereas the mean tumor size in our cohort was 15.2 mm consisting of CRLM, only, which again limits comparability [17, 32]. Although there are some differences in the 1-year LTP rate, the midterm results of our study with a 2-year LTP-free rate of almost 23% and a 3-year LTP-free rate of almost 20% are comparable with a previous study (2-year progression-free survival 18% [16]). To further evaluate and classify the advantages of IRE in the treatment of CRLM, especially after prior extended liver surgery, long-term clinical outcome data are needed.

We can further conclude that heterogeneity in target tumor size, origin and overall tumor biology, patient population, especially regarding prior liver surgery, and operator experience, have all been identified as potential confounders and are all potential causes for the reported high variability of local efficacy rates [32, 33].

Of the 12 patients with LTP 4 (33%) patients were diagnosed with tract seeding. Tract seeding is a known complication after IRE procedures in lung and liver [34, 35]. However, this is an instance that needs to be carefully avoided and is a clear disadvantage of the IRE method when compared to thermal ablation. Development of alternative application techniques such as trocars or alternative probe designs to limit the rate of tract seeding need to be pursued before a more widespread application of IRE can be recommended [36].

Still, where there is no other choice than palliative chemotherapy and/or best supportive care, because of the unfavorable location of the target tumor prohibiting (thermal) ablation and surgical resection, IRE is a relatively safe procedure and should be considered as a salvage treatment option.

There are several independent factors known to be associated with local tumor control after thermal ablation. In this study, there was no statistically significant association between patient age, tumor sidedness, size or synchronicity of CRLM.

This study suffers from limitations, such as its retrospective character and the small sample size. Furthermore, there was inhomogeneity in the colorectal cancer subtypes and the systemic therapies received pre-IRE, which may affect the response to IRE and the overall oncological outcome. Additionally, we did not compare IRE to other local ablation methods such as MWA, although the latter should only be applied with caution in the posthepatectomy liver remnant, because of the risk of unintended large volume ablation and potential damage to bile ducts [37].

## Conclusion

In conclusion, in this highly selected patient population with irresectable local recurrences of CRLM after major liver surgery, IRE was shown to be a safe salvage treatment option for high-risk tumor localization, when (thermal) ablation and/or resection is contraindicated. With IRE, a short- to midterm intrahepatic progression-free survival of 20% of can be achieved.
